# First case report of Cohen syndrome in the Tunisian population caused by *VPS13B* mutations

**DOI:** 10.1186/s12881-017-0493-5

**Published:** 2017-11-17

**Authors:** Imen Rejeb, Houweyda Jilani, Yasmina Elaribi, Syrine Hizem, Lamia Hila, Julia Lauer Zillahrdt, Jamel Chelly, Lamia Benjemaa

**Affiliations:** 1 Service des Maladies Congénitales et Héréditaires, CHU Mongi Slim La Marsa, Sidi Daoud La Marsa, 2046 Tunis, Tunisia; 2 Laboratoire de Génétique Humaine, Faculté de Médecine de Tunis, Tunis, Tunisia; 3 Institut Cochin, Université Paris-Descartes, CNRS (UMR 8104), Paris, France; 4 Inserm, U1016, Paris, France; 5 Pôle de biologie, Hôpitaux Universitaires de Strasbourg, Strasbourg, France

**Keywords:** Cohen syndrome, *VPS13B* gene, Compound heterozygous mutation

## Abstract

**Background:**

Cohen syndrome is a rare autosomal recessive developmental disorder that comprises variable clinical features counting developmental delay, pigmentary retinopathy, myopia, acquired microcephaly, truncal obesity, joint hypermobility, friendly disposition and intermittent neutropenia. *VPS13B* (vacuolar protein sorting 13, yeast, homologue of B) gene is the only gene responsible for Cohen Syndrome, causative mutations include nonsense, missense, indel and splice-site variants. The integrity of the Golgi apparatus requires the presence of the peripheral membrane protein *VPS13B* that have an essential function in intracellular protein transport and vesicle-mediated sorting.

**Case presentation:**

In this study, we performed whole exome sequencing (WES) in a Tunisian family with two young cases having developmental delay, hypotonia, autism spectrum disorder, ptosis and thick hair and eyebrows. The proposita presented also pigmentory retinopathy. Compound heterozygous mutation in *VPS13B* gene was detected by WES. This mutation inherited from healthy heterozygous parents, supports an unpredictable clinical diagnosis of Cohen Syndrome. The proband’s phenotype is explained by the presence of compound heterozygous mutations in the *VPS13B* gene*.* This finding refined the understanding of genotype-phenotype correlation.

**Conclusions:**

This is the first report of a Tunisian family with Cohen syndrome mutated in the *VPS13B* gene.

## Background

Cohen syndrome (CS) (MIM# 216550) is a rare autosomal recessive developmental disorder characterized by Cohen and colleagues in 1973 [1]. Truncal obesity, intellectual disability, developmental delay, joint laxity, craniofacial dysmorphism, high myopia and/or retinal dystrophy and neutropenia are typical clinical manifestations of the syndrome [1, 2].At present, CS has been essentially assigned to mutations in the *VPS13B* gene (MIM# 607817) among patients from diverse ethnicity. *VPS13B*, the single CS linked gene so far described, is localized on q22.2 locus of chromosome 8. Its length is about 864 kb and comprises 62 exons. The longest transcript [NM_017890.4] is 14,100 bp long encoding for a 4022 amino acid protein. *VPS13B* is a peripheral membrane protein with putative transmembrane domains and functional motifs that have an essential function in the transport of intracellular proteins and in vesicle-mediated sorting [3]. The expression of the VPS13B is mainly noticed in the whole body and in the central nervous system, blood, muscles, and heart [4]. Approximately, 200 cases of the CS and about more than 150 deleterious mutations have been identified to date (http://www.hgmd.org); in most cases mutations are stop codon mutations that result in a functionally null protein. The diagnosis is always difficult in childhood, this is due to the fact that many of the typical traits may be nonexistent till scholarisation or upcoming years and intermittent neutropenia is not consistently observable.

Here we report the characterization of a new compound heterozygous mutation in *VPS13B* gene in 2 Tunisian related cases with CS.

## Case presentation

The present study describes clinical and molecular findings in two patients with CS from a non-consanguineous Tunisian family. One of the authors examined the patients. DNA was extracted from peripheral blood using standard methods. The patients’ parents gave their consent.

The proposita was the first child of non-consanguineous and healthy condition parents. Pregnancy was normal and carried to term with an APGAR score of 9 and 10 at one and five minutes after birth, respectively. The parameters at birth were: weight 3300 g (60th percentile), height 50 cm (70 th percentile), occipitofrontal circumference (OFC) 35 cm (85th percentile). Hypotonia and poor sucking were noticed. She presented delayed psychomotor development: she wasn’t capable to sit without help till 12 months and walked at the age of 2 years. At 3 years, the patient was not capable to speak. Ophthalmological evaluation showed left strabismus and pigmentary retinopathy.

**Fig. 1:**
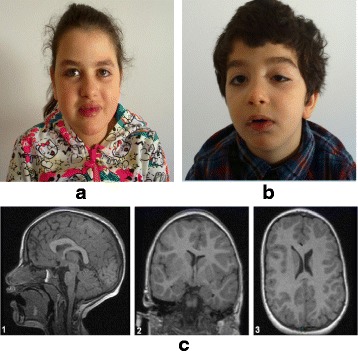
**a** The proband**. b** the brother of the proband**. c** Proband’s MRI (1) sections showing dysmorphic and thick corpus callosum and (2, 3) the lateral ventricular asymmetry

When reevaluated at 12 years, growth retardation and progressive microcephaly were noted. We noticed a weight of 31 kg (−1 SD), a height of 126 cm (−3.2 SD) and an OFC of 48 cm (−3.8SD). At neurological assessment, we noticed widespread hypotonia and joint hypermobility. Clinical diagnosis revealed dysmorphic facial features as thick hair eye brows and lashes, prominent upper central incisors, prominent lips and short philtrum; the last two features lead to a half open-mouth. The hands were small with tapering fingers. She also presented truncal obesity (Fig. 1). Communication and social skills were impaired. She presented intellectual disability with autistic-like traits.

Cerebral MRI showed thick and dysmorphic corpus callosum and lateral ventricular asymmetry. The brainstem and the cerebellum were normal (Fig. 1c). Karyotype and CGH array were normal.

Her brother presented at the age of 6 years a weight of 22 kg (normal), a height of 113 cm (normal) and an OFC of 48 cm (−3SD). Birth parameters were: weight 3350 g (50th percentile), height 50 cm (50th percentile), occipitofrontal circumference (OFC) 36 cm (85th percentile). Pregnancy was normal and carried to term with an APGAR score of 10 and 10 at one and five minutes after birth, respectively. He was born with a ptosis in the left eye operated at the age of 5 years (Fig. 1). He presented high myopia. He had, like his sister, thick hair eyebrows and lashes, he presented downslanted palpebral fissures, micrognathia, arched palate, clinodactyly of the toes, slender hands and feet and tapering fingers. Intellectual disability and stereotyped motor behavior were also noticed.

After the identification of the causal mutation, the reverse phenotyping showed moderate neutropenia and mild neutropenia in the proposita and her brother respectively. In fact, neutropenia is one of the important clinical signs of CS, but because of the nonexistence of clinical signs and the occasional occurrence, it is rarely identified.

The CARE guidelines were followed.

### Genetic testing

Because of limitations of renseignement on neutropenia and pigmentary retinopathy we didn’t notice that the clinical features matched with a CS, so we first use WES approach. We performed WES in trio made of the proband and his two unrelated parents.

Library generation, exome enrichment and whole-exome sequencing methodology used are detailed in the article written by Poirier et al. [5].

We analyzed variants affecting coding regions and essential splicing sites and excluded all variants with a frequency greater than 1% according to genomic databases (dbSNP, 1000 Genomes, Exome variant server and local platform database). All relevant variant were visually explored with Integrative Genomics Viewer (IGV: http://software.broadinstitute.org/software/igv/) to detect false positive results.

With this method and these filters, 9 variants were detected in index case (7 with de novo model of inheritance and 2 in the same gene with recessive model). 5 out of the 7 “de novo” variants appeared inherited from one parent (IGV), the 2 others were absent in the affected brother. The 2 variations in *VPS13B* gene (identified by recessive model of analysis) were also present in the brother; the c.3582delT, p.A1194fs were inherited from the mother and the c.6295_6296delAT, p.M2124 fs one from father.

Finally, we confirmed by PCR and Sanger sequencing of all coding regions and exon-intron boundaries of the *VPS13B* gene the relevant variants identified by WES. Using genomic DNA from the proband, her parents and her brother, the mutations identified were tested for familial segregation.

**Fig. 2:**
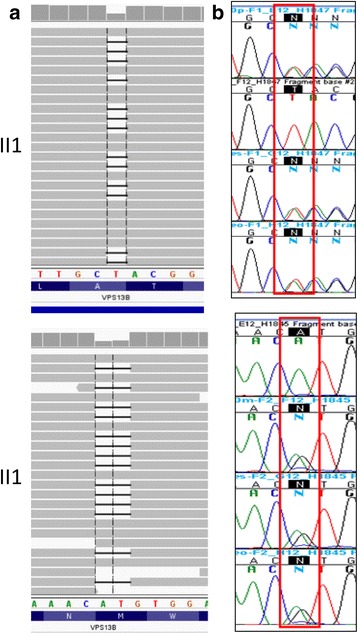
**a** Integrative Genomics Viewer of short read alignment indicated the compound heterozygous variant identified by exome sequencing (100,479,778 T/−,100712001AT/−) in the *VPS13B* gene. **b** Sanger validation shows the compound heterozygosity of the proband II1 formed by the c.3582delT mutation, and the c.6295_6296delAT mutation

WES of individual II1 noticed the presence of a compound heterozygous variant (100,479,778 T/−, 100712001AT/−) in the *VPS13B* gene (Fig. 2a). This compound heterozygous mutation in the *VPS13B* gene (NM_017890.4) was validated by Sanger sequencing (c.3582delT, p.A1149fs + c.6295_6296delAT, p.M2124 fs) (Fig. 2b) inherited from healthy heterozygous parents confirms the diagnosis of CS.

**Fig. 3:**
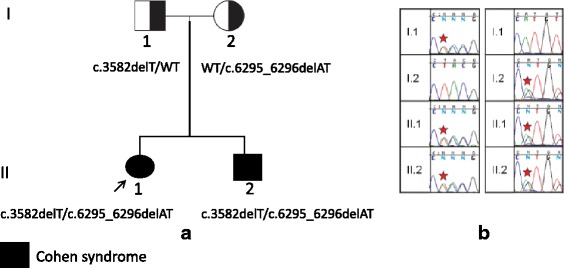
**a** Pedigree of the Tunisian family and compound heterozygosity of the proband, formed by the c.3582delT mutation, inherited from the father of the proband and also present in her brother, and the c.6295_6296delAT mutation inherited from the mother. The 2 mutations are shown in black, and in white the wild type alleles (WT). **b** Sanger validation shows segregation of the VPS13B mutations in the four family members

Sanger sequencing showed that the variations c.3582delT (p.A1149fs) and c.6295_6296delAT (p.M2124 fs) in the *VPS13B* gene present in both the proband and her brother were of biparental origin (Fig. 3).

## Discussion and conclusions

We report a Tunisian family including two siblings with developmental delay and intellectual disability harbouring a novel compound heterozygous mutation in the *VPS13B* gene by WES. Even for an experienced clinician, the diagnosis of CS is difficult. In fact this syndrome is a rare autosomal recessive disorder and it is often impossible to diagnosis CS until middle or late childhood. Therefore the phentotypic traits are very variable, several could be lacking till scholarisation or upcoming years. CS can be retained when six of eight phentotypic traits are noticed including developmental delay, joint hypermobility, typical CS facial gestalt, high myopia and/or retinal dystrophy, microcephaly, truncal obesity with slender extremities, overly sociable behaviour and neutropenia [6]. However some of these features cannot be observable till scholarisation or upcoming years, like retinal dystrophy, truncal obesity and overly sociable behavior, furthermore the typical facial gestalt of prominent incisors is always absent. In fact, when the girl was first seen at 7 years and the boy at 1 year, respectively, they had postnatal-onset microcephaly and delayed developmental milestones, we didn’t suspect the CS at this time given no other revealing traits.

Mutations in the *VPS13B* gene are responsible for CS. The novel compound heterozygous mutation in *VPS13B* inherited from healthy heterozygous parents, c.3582delT (p.A1149fs) and c.6295_6296delAT (p.M2124 fs) inherited respectively from the father and the mother are present in the proband and her brother causes a frameshift that induce a premature stop codon. Subsequently this frameshift mutation generate a premature stop codon that can either induce a truncated protein lacking a number of functional domains of the VPS13B protein or to a functional null-allele as a result of a non sense mediated mRNA decay (NMD).

Actually, approximately 188 mutations in *VPS13B* gene have been identified, 153 of them were associated with CS (http://www.hgmd.org/). *VPS13B* is a Golgi-associated peripheral membrane protein co-localizing with the cis-Golgi matrix protein GM130. RP2 and RPGR, two retinitis pigmentosa disease genes, situate to the Golgi, and depletion of RP2 causes abnormal Golgi function and protein transport in the photoreceptor [7, 8]. This highlights that a normal function of Golgi-associated proteins, including VPS13B, is essential for a good functioning of the photoreceptor. Recent studies have concluded that VPS13B mutations, responsible for COH1, are responsible of a tissue-specific major defect of glycosylation and endosomal–lysosomal trafficking defect. This highlights that VPS13B is essential in Golgi glycosylation and morphology, as well as in lysosomal–endosomal pathway maintenance [9].

Using WES in the early stage of disease for young patients where the clinical diagnosis is not evident is actually the best approach. Currently, the exome sequencing approaches are used in many laboratories helping clinicians in solving the aetiology of many rare diseases. This approach, compared to sanger sequencing helps saving costs and time especially for large genes such as *VPS13B* gene. In fact, the costs of sequencing per base with sanger sequencing is much higher than with NGS [10, 11].So, sanger sequencing will be performed only if a causative mutation will be identified by NGS in order to validate this variant. Therefore, for mendelian diseases, especially for those with genetic heterogeneity, NGS is almost certainly the best primary choice in genetic tests.

In conclusion, we report the first Tunisian family with CS, a novel compound heterozygous mutation in *VPS13B* gene, identified using WES is the deleterious mutation in the patients of this family.

## Abbreviations

APGAR: Appearance pulse grimace activity and respiration
CGH: Comparative genomic hybridization
CS: Cohen syndrome
MRI: Magnetic resonance imaging
NGS: Next-Generation Sequencing
NMD: Nonsense-mediated mRNA decay
OFC: Occipitofrontal circumference
PCR: Polymerase chain reaction
WES: Whole exome sequencing
